# ICTV Virus Taxonomy Profile: Rhabdoviridae 2026

**DOI:** 10.1099/jgv.0.002255

**Published:** 2026-06-09

**Authors:** Peter J. Walker, Nicolas Bejerman, Kim R. Blasdell, Humberto Debat, Ralf G. Dietzgen, Anthony R. Fooks, Juliana Freitas-Astúa, Kyle Garver, Hideki Kondo, Pedro Luis Ramos-González, Mang Shi, Robert B. Tesh, Noël Tordo, Nikos Vasilakis, Anna E. Whitfield

**Affiliations:** 1 University of Queensland, St Lucia, QLD 4072, Australia; 2 Consejo Nacional de Investigaciones and Instituto Nacional de Tecnología Agropecuaria (INTA), Buenos Aires, Argentina; 3 CSIRO Health and Biosecurity, Geelong, VIC 3220, Australia; 4 Animal and Plant Health Agency Addlestone, Surrey KT15 3NB, UK; 5 Brazilian Agricultural Research Corporation, Cruz das Almas-BA, 44380-000, Brazil; 6 Fisheries & Oceans Canada, Nanaimo, V9T 6N7 BC, Canada; 7 Okayama University, Kurashiki, 710-0046, Japan; 8 Instituto Biológico, São Paulo, Brazil; 9 Sun Yat Sen University, Guangzhou, Guangdong, PR China; 10 University of Texas Medical Branch, Galveston, TX 77555, USA; 11 Gamal Abdel Nasser University, Conakry, Guinea; 12 North Carolina State University, Raleigh NC 27606, USA

**Keywords:** ICTV Report, *Rhabdoviridae*, taxonomy

## Abstract

The family *Rhabdoviridae* comprises viruses with unsegmented, bi-segmented or tri-segmented negative-sense (−) RNA genomes of 10–16 kb. Virions are typically enveloped, with bullet-shaped or bacilliform morphology, but can also be non-enveloped filaments. Rhabdoviruses infect plants or animals, including vertebrates or invertebrates such as arthropods, which can serve as single hosts or act as biological vectors for transmission to animals or plants. Rhabdoviruses include important pathogens of humans, livestock, fish or agricultural crops. This is a summary of the International Committee on Taxonomy of Viruses (ICTV) Report on the family *Rhabdoviridae*, which is available at ictv.global/report/rhabdoviridae.

## Virion

**Fig. 1.**
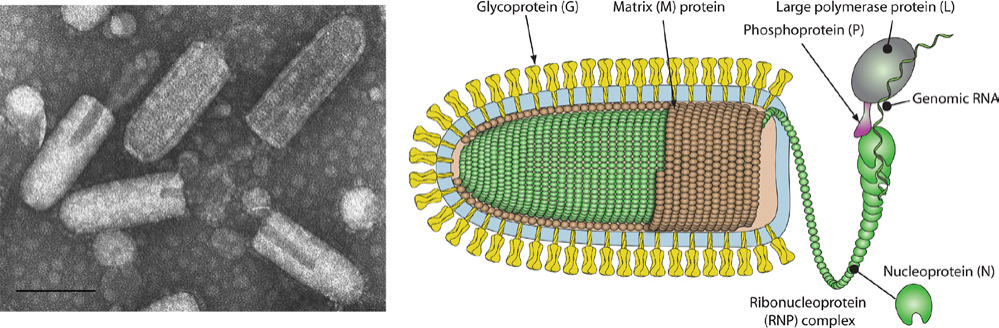
(Left) Negative-contrast electron micrograph of vesicular stomatitis Indiana virus particles. Bar: 100 nm (courtesy of P. Perrin). (Right) Schematic illustration of a rhabdovirus virion and ribonucleocapsid structure (courtesy of P. Le Mercier).

Virions are usually enveloped and bullet-shaped or bacilliform (i.e. with two rounded ends) and contain five structural proteins (Table 1, Fig. 1). The nucleocapsid protein (N), the large multi-functional RNA-directed RNA polymerase (L) and the polymerase-associated phosphoprotein (P), together with the RNA genome, form the ribonucleoprotein (RNP) complex. The nucleocapsid is encased in the matrix protein (M) layer, which also interacts with the envelope containing the transmembrane glycoprotein (G). Rhabdoviruses assigned to the genera *Dichorhavirus* and *Varicosavirus* lack envelopes. Many rhabdoviruses have been identified only as metatranscriptomic sequences, with no virus isolate available.

**Table 1.** Characteristics of members of the family *Rhabdoviridae*

Example	vesicular stomatitis Indiana virus (AF473864), species *Vesiculovirus indiana*
Virion	Bullet-shaped or bacilliform particle, 100–430 nm in length and 45–100 nm in diameter, composed of a helical nucleocapsid surrounded by a matrix layer and a lipid envelope. Some rhabdoviruses have non-enveloped filamentous virions
Genome	Negative-sense RNA of 9.8–16.3 kb (unsegmented, bi-segmented or tri-segmented)
Replication	RNP complexes containing anti-genomic RNA are generated and are templates for the synthesis of RNP complexes containing genomic RNA
Translation	From capped and polyadenylated mRNAs transcribed progressively from each gene (3′ to 5′), sometimes containing multiple ORFs
Host range	Vertebrates, invertebrates and plants; many vertebrate and plant rhabdoviruses are arthropod-borne
Taxonomy	Realm *Riboviria*, kingdom *Orthornavirae*, phylum *Negarnaviricota*, subphylum *Haploviricotina*, class *Monjiviricetes*, order *Mononegavirales*: >3 subfamilies, >63 genera, >610 species

## Genome

**Fig. 2.**
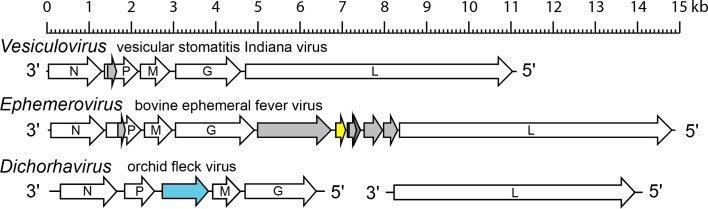
Schematic representation of several rhabdovirus genome organizations. ORFs encoding the viroporin (yellow) and movement proteins (blue) are shown.

Rhabdovirus negative-sense (−) RNA genomes are 10–16 kb (Fig. 2) [1]. Almost all rhabdovirus genomes are unsegmented, but some have bi- or tri-segmented genomes [2,3]. Terminal non-coding regions are partially complementary. Genomes usually encode five major structural proteins but may lack the gene encoding the transmembrane G, and may encode additional (accessory) proteins as alternative ORFs within the structural protein genes or in additional genes [1,4]. These commonly include genes encoding class I viroporins [5]. Plant rhabdoviruses contain an additional structural protein, P3, responsible for cell-to-cell movement.

## Replication

Rhabdovirus replication generally occurs in the cytoplasm following receptor-mediated endocytosis. Primary transcription is initiated from the incoming (−) RNP complex by the RNA-directed RNA polymerase (RdRP) [6]. Stop–start transcription occurs from 3′ to 5′ using gene start and gene end sequences to generate polyadenylated mRNAs. Replication is initiated by the RdRP from a single promoter at the 3′-end, ignoring gene start and gene end sequences to generate a (+) RNP. This is the template for nascent (−) RNPs, which are assembled with M and G into enveloped virions . Budding can occur at either the plasma membrane or internal membranes. Some plant rhabdoviruses replicate in the nucleus. Rhabdoviruses include important pathogens of humans, livestock, fish or agricultural crops.

## Taxonomy

Current taxonomy: ictv.global/taxonomy. Each genus forms a monophyletic clade based on phylogenetic analyses of virus L-protein sequences and members usually have similar ecology and genome architecture [1,4, 7]. Viruses in the subfamily *Alpharhabdovirinae* (>35 genera) infect vertebrates (mammals, birds, reptiles, amphibians) or invertebrates, or vertebrates and arthropod vectors. Viruses in the subfamily *Betarhabdovirinae* (>11 genera) infect plants and arthropod vectors; some have bi- or tri-segmented genomes (genera *Dichorhavirus*, *Varicosavirus* and *Trirhavirus*), rod-shaped, non-enveloped virions (genus *Varicosavirus*) or are transmitted by fungi. Hosts for viruses in the subfamily *Gammarhabdovirinae* are teleost fish or molluscs, and for the subfamily *Deltarhabdovirinae*, insects, ticks, crustaceans and nematodes. Viruses in genera not assigned to a subfamily have been detected by high-throughput sequencing of platyhelminths or vertebrate tissues.

## Resources

Full ICTV Report on the family *Rhabdoviridae* is available at ictv.global/report/rhabdoviridae.

## Abbreviations

G: glycoprotein
ICTV: International Committee on Taxonomy of Viruses
L: large polymerase protein
M: matrix protein
N: nucleocapsid protein
P: phosphoprotein
RdRP: RNA-directed RNA polymerase
RNP: ribonucleoprotein
